# Serological and molecular epidemiology of Japanese Encephalitis in Zhejiang, China, 2015-2018

**DOI:** 10.1371/journal.pntd.0008574

**Published:** 2020-08-27

**Authors:** Xuan Deng, Ju-ying Yan, Han-qing He, Rui Yan, Yi Sun, Xue-wen Tang, Yang Zhou, Jun-hang Pan, Hai-yan Mao, Yan-jun Zhang, Hua-kun Lv

**Affiliations:** Zhejiang Provincial Center for Disease Control and Prevention, Hangzhou, People’s Republic of China; International Vaccine Institute, REPUBLIC OF KOREA

## Abstract

**Background:**

Shifts have occurred in the epidemiological characteristics of Japanese encephalitis (JE), extending from the molecular level to the population level. The aim of this study was to investigate the seroprevalence of JE neutralizing antibodies in healthy populations from different age groups in Zhejiang Province, and to conduct mosquito monitoring to evaluate the infection rate of Japanese encephalitis virus (JEV) among vectors, as well as the molecular characteristics of the E gene of isolated JEV strains.

**Methodology/Principal findings:**

A total of 1190 sera samples were screened by a microseroneutralization test, including 429 infants (28d-11m) and 761 participants (2y-82y). For those under 1 year old, the geometric mean titers (GMTs) of the JE neutralizing antibody was 9.49 at birth and significantly declined as the age of month increased (r = -0.225, *P*<0.001). For those above 1-year old, seropositive proportions were higher in subjects aged 1–3 years old as well as ≥25 years old (65%-75%), and relatively lower in subjects aged between 4–25 years old (22%-55%). Four or more years after the 2^nd^ dose of JEV-L (first dose administered at 8 months and the second at 2 years of age), the seropositive proportion decreased to 32.5%, and GMTs decreased to 8.08. A total of 87,201 mosquitoes were collected from livestock sheds in 6 surveillance sites during 2015–2018, from which 139 E gene sequences were successfully amplified. The annual infection rate according to bias-corrected maximum likelihood estimation of JEV in *Culex tritaeniorhynchus* was 1.56, 2.36, 5.65 and 1.77 per 1000, respectively. JEV strains isolated during 2015–2018 all belonged to Genotype I. The E gene of amplified 139 samples differed from the JEV-L vaccine strain at fourteen amino acid residues, including the eight key residues related to virulence and virus attenuation. No divergence was observed at the sites related to antigenicity.

**Conclusions/Significance:**

Zhejiang Province was at a high risk of JE exposure due to relatively lower neutralizing antibody levels among the younger-aged population and higher infection rates of JEV in mosquitoes. Continuous, timely and full coverage of JE vaccination are essential, as well as the separation of human living areas and livestock shed areas. In addition, annual mosquito surveillance and periodic antibody level monitoring are important for providing evidence for improvement in JE vaccines and immunization schedules.

## Author summary

Although Japanese encephalitis (JE) has been well-controlled in Zhejiang Province, it remains a hot public health issue due to heavy disease burden. The epidemiological characteristics of JE have changed recently in Zhejiang Province. Increasing proportion of adult cases (>40 years old) have been reported. Genotype I (GI) of JEV has displaced genotype III (GIII) as the dominant genotype. The population immunity against JE was notably low among participants aged 4–25 years old, with the lowest GMT being in the 7-14-year-old group. Two doses of JEV-L did not provide effective protection after 4 or more years past the 2^nd^ dose. Therefore, issues about duration of protection and necessity of a booster dose at 6 years of age need further research. JEV strains isolated from mosquitoes during 2015–2018 in Zhejiang Province all belonged to GI. Compared with the JE vaccine strain, mutations at the eight amino acid residues on E gene related to virulence in Zhejiang strains were detected, while the antigenic sites remained the same. JE serological survey in healthy populations with different sequential immunization schedule should be conducted to provide evidence for reformation on JE immunization strategy, including type, dose and interval.

## Introduction

Japanese encephalitis (JE), caused by Japanese encephalitis virus (JEV), is one of the most serious vector-borne viral encephalitis in Southeast Asia, Western Pacific Region, and Northern Australia [1–2]. A mixed infection with yellow fever was also identified in Africa [3]. Approximately 3 billion people are exposed to the risk of JEV infection. It is estimated that 67,900 JE cases occur annually in 24 JE-endemic countries, with an overall incidence of 1.8 per 100,000 [4]. Although symptomatic Japanese encephalitis is rare, and only approximately 1 in 250 infections results in severe clinical symptoms, the case fatality rate can be as high as 30%. Permanent neurological or psychiatric sequelae can occur in 30%–50% of survivors, resulting in heavy health, social and economic burdens [1].

JEV is maintained in a natural transmission cycle involving mosquito vectors and amplifying vertebrate hosts, such as pigs and wading birds. JEV can proliferate in reservoirs, leading to a longer period of viremia and a higher viral load than in humans, who are identified as a dead-end host for JEV [5]. Mosquitoes, especially *Culex tritaeniorhynchus* in China, play an important role in JEV transmission chain. The mosquitoes become infected through biting of JEV reservoirs and then transmit JEV to humans through another bite. JEV belongs to the genus *Flavivirus*, family *Flaviviridae* with only one serogroup. The JEV virion contains three structural proteins: nucleocapsid or core protein (C), non-glycosylated membrane protein (M), and glycosylated envelope protein (E). X-ray crystallography revealed that the E protein was composed of three distinct domains (I to III). Past studies [6–9] have confirmed that the E protein is responsible for neurovirulence and is involved in many important biological processes, including viral attachment, fusion, penetration, hemagglutination, virus neutralization, host range and cell tropism. According to the nucleotide sequence of E gene, JEV can be divided into five genotypes, I to V. Xiaoyan Gao conducted phylogenetic analysis on the whole genomic sequences of all the five genotypes and found that the ancestral lineage diverged in the order V, IV, III, II, and I[10]. The earliest reserved isolated JEV strain in China was derived from a viral encephalitis patient in Beijing, 1949, which belongs to GIII. During the routine monitoring in China, GI (first in 1979, Yunnan) and GV (first in 2009, Tibet) were successively discovered. The evolutionary trend of JEV genotype in China changes from GIII-only (1949–1971) to GIII+GI- coexist (1979–2004), and then to GI-dominant (2005-now) [2,10,11]. Recent studies [12–15] also show that GI is gradually replacing GIII and becoming the dominant type circulating in Asia. However, GV, which was first isolated in Malaysia in 1952 and has not been detected over the last 57 years, reemerged in two areas, and both are isolated from *Culex tritaeniorhynchus*: Tibet (2009) and South Korea (2010) [11]. A recent study [16,17] showed that the current JE vaccine induced low protective immunity against the emerging JEV GV. Therefore, changing geographic distribution of JEV genotypes brings new challenges for JE prevention and control.

Zhejiang Province, which belongs to the subtropical monsoon climate, used to be JE endemic area, with the highest incidence of 47.5/100,000 in 1967 [18]. With the widespread use of vaccines, the rapid development of economy and remarkable improvement of sanitary conditions, the JE incidence had decreased sharply after the 1970s. Fast review of JE vaccine application in Zhejiang Province, Inactivated Mouse Brain–Derived JE vaccine (MBD JEV, P3 strain) was first used in 1953, and then Inactivated Primary Hamster Kidney cell-derived JE vaccine was adopted in the 1970s. In 1989, live attenuated JE vaccine (JEV-L, SA 14-14-2 strain) was introduced and quickly became widespread. With the implementation of the National Expanded Program on Immunization (EPI) in 2008, two doses of JEV-L were required at 8 months and 2 years of age, respectively [19].

In addition to vaccination, JE surveillance in Zhejiang Province, including mosquito surveillance, antibody level surveillance in humans and pigs, acute meningitis and encephalitis syndrome surveillance (AMES), also have an important impact on JE prevention and control. Mosquito surveillance, which is the earliest and longest-running monitoring program (1982–1983, 2006–2019) in Zhejiang Province, provides important evidence for the natural infection rate and genotypes of JEV in mosquito vectors. As early as in 1982, JEV (GIII) was first isolated from *Culex tritaeniorhynchus* collected from pigpens and cowsheds in Dinghai County and Yiwu County. However, the JEV genotype has been replaced by GI since 2006 in all the following surveillance sites. Antibody level surveillance in healthy people was conducted from 2006–2016 (except for 2010), indicating the immunity barriers and herd immunity of the target population. Antibody level surveillance in pigs was conducted from 2006 to 2013, providing evidence for natural infection rate of JEV in reservoirs. However, with the integration and reduction of pig farms due to swine fever, sample collection from young piglets was considerably more difficult, and the surveillance program ended in 2014. AMES was conducted in Xianju County from 2006–2015 which reflected the true proportion of reported JE cases and explored the epidemiology and pathogenic spectrum of AMES. Although the surveillance program stopped in 2016, follow-up might be restarted.

Zhejiang had accumulated a degree of experience in JE surveillance. To provide guide and evidence for improvement in JE vaccines and the JE immunization strategy, we analyzed and evaluated the results of surveillance programs, including mosquito surveillance and antibody level surveillance in healthy people, identified the JEV genotypes circulating in Zhejiang Province during 2015–2018 and analyzed the molecular characteristics of E gene.

## Materials and methods

### Epidemiology of JE in Zhejiang Province, 2015–2018

The data of morbidity and mortality of JE in Zhejiang Province were collected from National Notifiable Disease Registry System (NNDRS) of China and case based JE surveillance system (JESS) from 2015 to 2018. Descriptive epidemiological method was used to analyze.

### Antibody level surveillance in healthy people

#### Serum collection

A cross-sectional serological investigation was carried out in Jinhua city of Zhejiang Province in 2015–2016. The sample size for the survey was calculated by Epi Info [20], which is a public domain tools for epidemiological statistics. With the expected frequency set as 80%, confidence limits as 5%, design effect for cluster surveys as 2, and sample size was 492 with the confidence level as 95%. According to the JE vaccine immunization schedule and the epidemiological characteristics of JE in Zhejiang Province, participants were divided into twenty age groups (every month age was a group for under 1 year old, 1–3 years, 4–6 years, 7–14 years, 15–19 years, 20–24 years, 25–34 years, 35–44 years, 45 years and above). Sampling sites included maternity and pediatric hospital, kindergarten, school, community health service center, factory, and company. Combined with the annual physical examination, participants were requested to collect a 2ml-blood sample by qualified nurses, which would be stored in refrigerated containers (2–8°C) temporarily and transferred to -70°C before testing.

The study protocol was approved by the ethics committee of Zhejiang Provincial Center for Disease Control and Prevention. Written informed consent was obtained from all participants or legal guardians of the subjects before enrollment.

#### Micro-neutralization assay

Micro-neutralization assay was used to measure the presence of neutralizing antibodies against JEV. The procedure of micro-neutralization assay has been described elsewhere in detail [18]. The tests were conducted in Biological Safety Level 2 JE Laboratory of Zhejiang Provincial Center for Disease Control and Prevention, which joined the national JE lab-net in 2011. P3(GIII) strain was neutralized by serum samples from participants, which was obtained from the National Institution for Food and Drug Control, China. Seropositive level of JE was defined as a titer level of ≥10 (1/dil). Values below 10 were defined as 5 for the purpose of calculations.

### Mosquito surveillance

#### Mosquito collection

**Fig 1:**
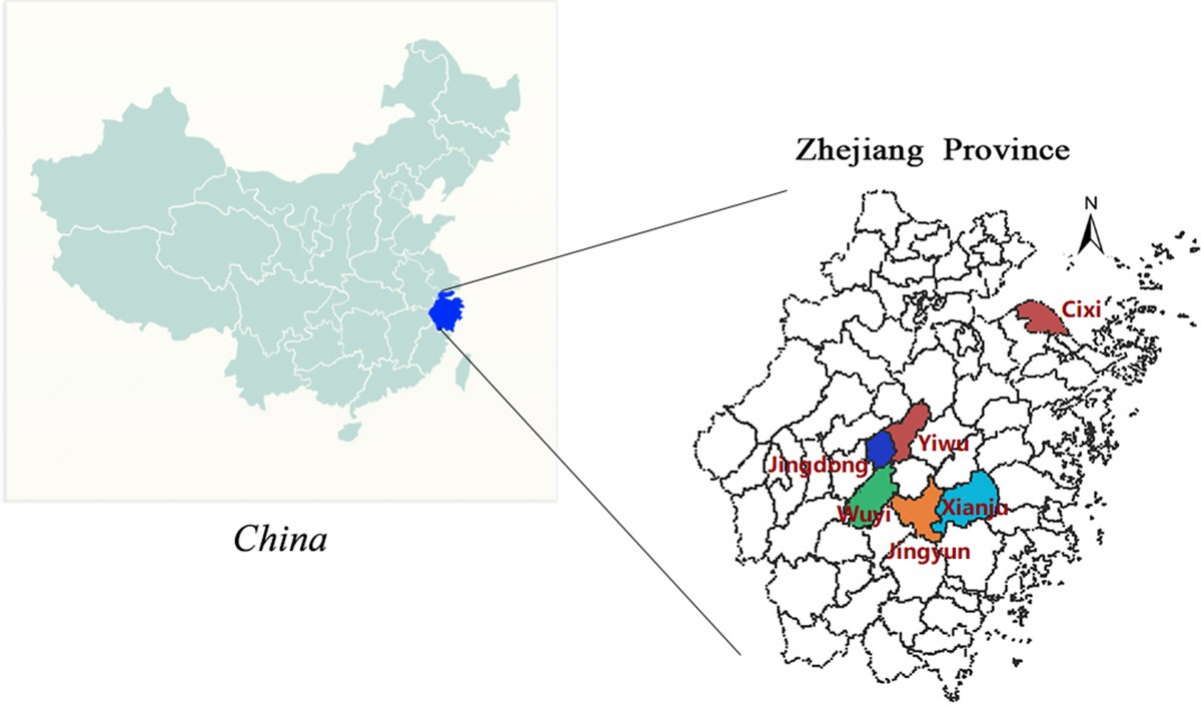
Sites of mosquito surveillance in Zhejiang Province, China, 2015–2018. Map was created by R version 3.5.0.

Mosquitoes were collected in pigpens and human dwelling from six counties in Zhejiang Province successively (Yiwu, Xianju, Jindong, Cixi, Jinyun and Wuyi) during June-August from 2015–2018 (Fig 1). Permissions were obtained from owners/residents of colleting sites. These sites both have pig farms and rice paddy fields, which are essential factors for JEV maintenance. At least 8 hand-held aspirators and 12–16 light-traps per site were used to collect mosquitoes after sunset (18:30–21:00). Mosquitoes were classified according to morphological characteristics [21], pooled (50–100 female individuals per pool) by species, date, site and sampling method. Mosquitoes containing blood were excluded in case of the intervention from pig blood. Specimens were stored in liquid nitrogen during transportation and transferred to -80°C until processing for virus isolation.

#### RNA extraction and polymerase chain reaction (PCR)

Pooled mosquitoes from -80°C were rapidly and fully grinded with 1.5 ml Hanks fluid and 1.5 ml grinding fluid. Then the homogenized mixture was centrifuged (12,000 r/min, 30min) to obtain the clarified supernatants, which was inoculated onto a monolayer of BHK-21 cells from the Chinese Center for Disease Control and Prevention, followed by cultured in a 5% CO_2_ humidity chamber at 37°C. Observe the cells daily to check the development of cytopathic effects (CPE). Those caused regular CPE in successive cell passages were regarded as virus positive.

Viral RNA was extracted from 200μl supernatant from virus-infected BHK-21 cell cultures using the RNeasy mini kit (QIAGEN, Hilden, Germany). Reverse transcription PCR (RT-PCR) was performed by TaKaRa One Step RNA PCR kit (TaKaRa Bio Inc, Dalian, China). JEV RNA was detected by quantitative real-time PCR (qRT-PCR) [22]. The primers JE955f (5’-TGYTGGTCGCTCCGGCTTA-3’) and JE2536r (5’-AAGATGCCACTTCCACAYCTC-3’) were used to amplify the 1500-nt E gene. The PCR reaction conditions have been described elsewhere in detail [18].5μl of amplified PCR products of E gene were sequenced by Sangon Biotech Co., Ltd.(Shanghai, China) [23]. Sequencing assembly and verification were conducted using DNASTA.lasergene. v7.1. Sequence alignment and homologous comparison of nucleotide and amino acid sequences were conducted using MEGA 7.0 and DNAman7 (Lynnon Biosoft), respectively.

#### Phylogenetic tree construction

Sequences of JEV PCR products in Zhejiang province were compared with other relevant strains obtained from GenBank database. The reference sequence MVEV-51 NC000943 was used as an out-group. Phylogenetic tree was constructed by MEGA 7.0 software, using the maximum likelihood method based on the General Time Reversible model. Bootstrap method was used to conduct phylogeny test with 1,000 bootstrap replications. The scale bar represents nucleotide substitutions per site.

#### Infection rate calculation

Infection rates were calculated by bias-corrected maximum likelihood estimation (MLE) using the Excel add-in PooledInfRate v.4 statistical software package [24] and expressed as the number of infected mosquitoes per 1000 individuals.

### Statistical analysis

The chi-square test or Fisher’s exact test was used to analyze the significance of JEV neutralizing antibody seropositive proportions against P3 strain among different age groups. The Kruskal-Wallis test was conducted to compare the GMTs of neutralizing antibodies among different age groups. Multiple pairwise comparisons were conducted using Nemenyi test. The Cochran-Armitage trend test was used to assess the association between rates with increasing age or time-interval. Spearman correlation tests were conducted to assess the association between GMTs with increasing age or time-interval.

## Results

### Epidemiology of JE in Zhejiang Province, 2015–2018

A total of 53 JE cases were reported in Zhejiang Province during 2015–2018, of which 28 (52.83%) were migrations from other provinces in China and acquired infections in Zhejiang. The annual incidence of JE ranged from 0.016/100,000 to 0.028/100,000, with an average annual incidence of 0.024/100,000. Only 1 case died in 2018 with an average annual mortality rate of 0.00045/100,000 and a case fatality rate of 1.89%. The occurrence of JE in Zhejiang Province had an obvious peak season (from June to August), and 37 cases (69.81%) developed illness in July. The mean age was 17.9 years old, ranging from 7 months to 60.3 years old. 29 cases (54.72%) were less than 15 years old. The highest average age-specific incidence was 0.109/100,000 for the 0-4-year-old group, followed by the 10-14-year-old group (0.094/100,000) and the 5-9-year-old group (0.088/100,000). The proportions of cases < 15 years old in 2015–2018 were 77.78%, 66.67%, 23.08% and 56.25% respectively. While the proportions of cases ≥ 40 years old were 0%, 6.67%, 23.08% and 12.50% respectively. According to the immunization information system, 13 cases (24.53%) had a clear immunization history of JE vaccine, 6 of which had one dose of JEV-L and 7 of which had two doses of JEV-L. The follow-up conducted 6 months after onset indicated that 31 cases (58.49%) were totally cured and 15 cases (28.30%) remained long-term sequelae, including cognitive impairment and neurological sequelae.

### Antibody level surveillance in healthy people

A total of 429 infants (under 1 year old) and 761 participants (over 1 year old) were enrolled between Jan 2015 and Sep 2016; from each of them, a 2ml blood sample was drawn and tested successfully. Participants were sampled from maternity and pediatric hospital (429 participants), kindergarten (118), school (197), community health service center (89), factory (186), and company (171). The overall GMTs of JE neutralizing antibody against the P3 strain for participants under 1 year old was 6.36, with a seropositive proportion of 16.08% (Table 1). The overall GMTs against the P3 strain for participants over 1 year old was 13.60, with seropositive proportions of 55.72% (Table 2).

**Table 1:** Japanese encephalitis neutralizing antibody and seropositive proportion against P3 strain for participants under 1 year old in Zhejiang, 2015–2016.

Age Group (m)	No.	P3 GMT (95%CI)	No. of P3 Seropositive	P3 Seropositive Proportion(%)
**<1**	58	9.49 (7.78, 11.56)	22	37.93
**1**	57	7.31 (6.05, 8.85)	14	24.56
**2**	52	6.17 (5.29, 7.2)	7	13.46
**3**	53	5.64 (5.04, 6.31)	5	9.43
**4**	33	5.99 (4.98, 7.2)	4	12.12
**5**	29	6.23 (5.02, 7.73)	4	13.79
**6**	23	6.30 (4.9, 8.09)	3	13.04
**7**	23	6.30 (4.89, 8.11)	3	13.04
**8**	24	5.81 (4.75, 7.11)	2	8.33
**9**	24	5.26 (4.81, 5.75)	1	4.17
**10**	29	5.53 (4.86, 6.29)	2	6.90
**11**	24	5.70 (4.76, 6.82)	2	8.33
**Total**	**429**	**6.36 (6.01, 6.73)**	**69**	**16.08**

Note: No. means the number of participants in the corresponding category; P3 means P3 strain of JEV; No. of P3 Seropositive means the number of participants who were seropositive against P3 strain; P3 seropositive proportion (%) means the proportion of participants with seropositive antibody level against P3 strain.

**Table 2:** Japanese encephalitis neutralizing antibody and seropositive proportion against P3 strain for participants above 1 year old in Zhejiang, 2015–2016.

Age Group (y)	No.	P3 GMT (95%CI)	No. of P3 Seropositive	P3 Seropositive Proportion (%)
**1–3**	89	19.29 (15.01, 24.79)	58	65.17
**4–6**	118	12.18 (10.29, 14.42)	65	55.08
**7–14**	104	6.89 (6.08, 7.81)	23	22.12
**15–19**	93	12.55 (9.92, 15.88)	41	44.09
**20–24**	83	12.18 (9.55, 15.53)	39	46.99
**25–34**	88	15.48 (12.63, 18.98)	57	64.77
**35–44**	80	19.68 (15.41, 25.15)	62	77.50
**≥45**	106	17.46 (14.78, 20.62)	79	74.53
**Total**	**761**	**13.60 (12.62, 14.65)**	**424**	**55.72**

Note: No. means the number of participants in the corresponding category; P3 means P3 strain of JEV; No. of P3 Seropositive means the number of participants who were seropositive against P3 strain. P3 Seropositive proportion means the proportion of participants with seropositive antibody level against P3 strain.

### GMTs and seropositive proportions of JE neutralizing antibody

**Fig 2:**
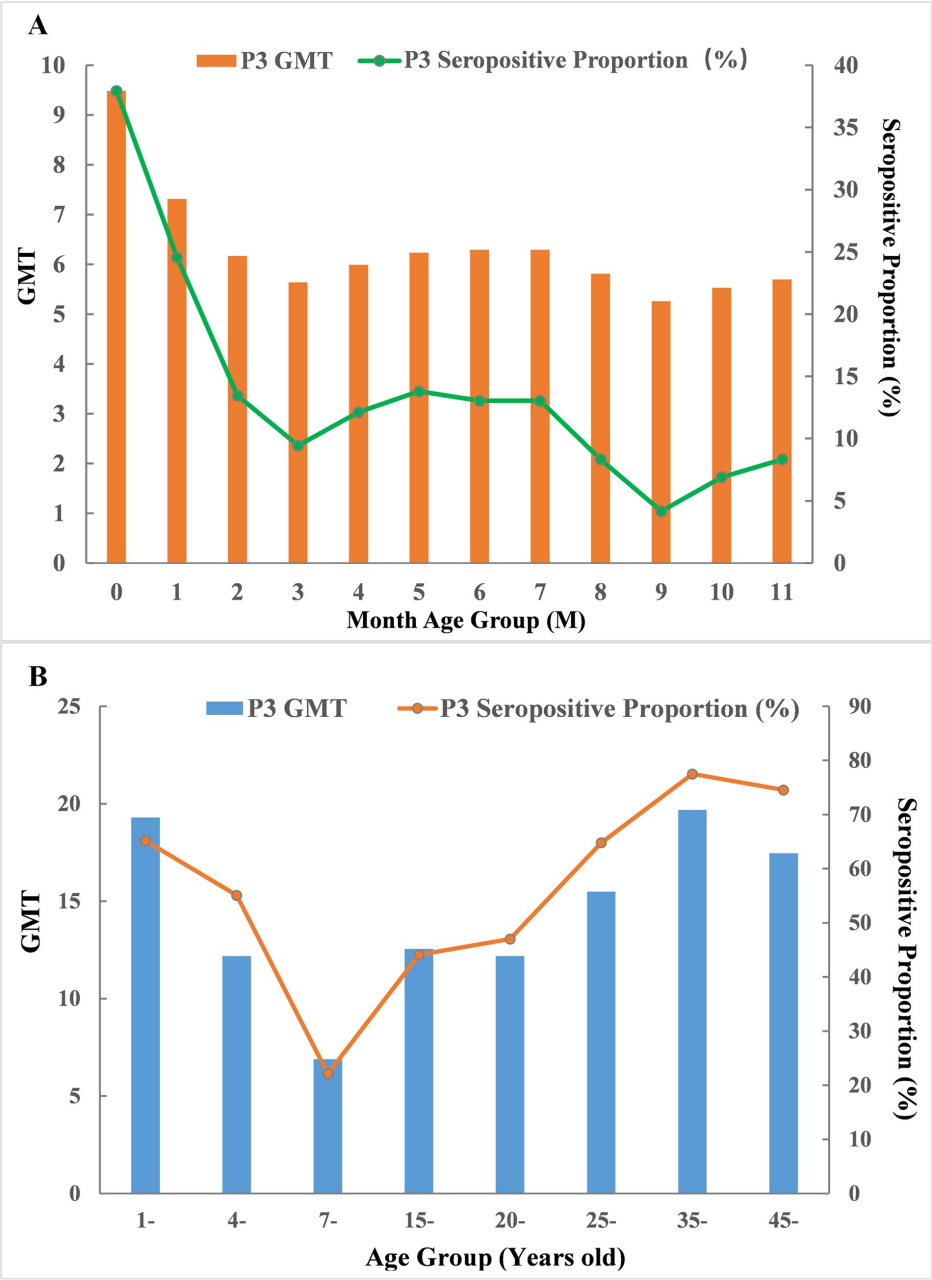
Geometric mean titers and seropositive proportion of Japanese encephalitis neutralizing antibody for different age groups (A: <1-year-old; B: >1-year-old group) in Zhejiang, 2015–2016.

A total of 429 participants under 1 year old were divided into 12 groups according to the month age (Table 1, Fig 2A). The GMT and seropositive proportion of the 9-month age group were both the lowest (5.26, 4.17%), while those of the <1-month age group were both the highest (9.49, 37.93%). Through Kruskal-Wallis test, significant difference was detected in GMT among all the 12 groups (*χ*^2^ = 31.25, *P* = 0.001). Further Nemenyi tests for multiple pairwise comparisons indicated that significant differences were only detected between the <1-month age group and the other three groups: 3 months of age, 9 months of age and 10 months of age (*P*_*1*_ = 0.004, *P*_*2*_ = 0.012, *P*_*3*_ = 0.018). Although significance differences in seropositive proportions were found among all the 12 groups using Fisher’s exact test (*P* = 0.003), only 2 groups (<1-month age group and 3-month age group) showed significant differences using the adjusted Chi-square test (*χ*^2^ = 12.22, *P*<0.001). The Spearman correlation test indicated that as the age of month increased, the GMT significantly declined (*r* = -0.225, *P*<0.001). The Cochran-Armitage trend test also supported the conclusion that the seropositive proportion decreased with increasing month age (*P*<0.0001).

A total of 761 participants above 1 year old were divided into 8 groups according to the JE vaccine immunization schedule and the epidemiological characteristics of JE in Zhejiang Province (Table 2, Fig 2B). The GMT and seropositive proportion against P3 strain of the 7-14-year-old group were both the lowest (6.89, 22.12%), while the 35-44-year-old group was the highest (19.68, 77.50%). The trends of the two indexes were similar in the age-group distributions: first declined rapidly to the lowest value at 7–14 years old and then increased slowly until 35 years old. Multiple pairwise comparisons indicated that the overall significant differences in GMT against P3 strain were mainly due to the 7-14-year-old group with other groups (*P*≤0.01 for all) and the 35-44-year-old group with the other three groups (4-6-year-old, 15-19-year-old, 20-24-year-old groups; *P*<0.05 for all).

### Trend of GMT and seropositive proportion after 2^nd^ vaccination

Among the 761 participants above 1 year old, 231 (30.35%) had a clear history of immunization on JE vaccine according to the immunization information system in Zhejiang Province, 219 of whom had two doses of JEV-L. According to the time interval between the 2^nd^ dose and date of blood drawn, 219 participants were divided into 5 groups (0–1 year, 1–2 years, 2–3 years, 3–4 years, and ≥4 years) (Table 3). Through Spearman correlation test, the GMTs against P3 strains declined as the time interval increased (*r* = -0.304, *P*<0.001), and dropped to below the threshold level of protection (1:10) after 4 or more years past the 2^nd^ dose. Cochran-Armitage trend test indicated that the seropositive proportions against P3 strains significantly decreased with increasing time interval (*P* = 0.0006), and dropped to less than 50% more than 4 years after the 2^nd^ dose.

**Table 3:** Japanese encephalitis neutralizing antibody and seropositive proportion against P3 strain for participants with two doses of JEV-L in Zhejiang, 2015–2016.

Years since the last dose	No.	P3 GMT (95%CI)	No. of P3 Seropositive	P3 Seropositive proportion(%)
**<1**	40	23.33 (15.79, 34.46)	28	70.00
**1-**	49	16.94 (12.4, 23.13)	32	65.31
**2-**	36	15.48 (11.05, 21.69)	23	63.89
**3-**	54	11.7 (9.32, 14.69)	30	55.56
**4-**	40	8.08 (6.29, 10.39)	13	32.50
**Total**	**219**	**14.01 (12.17, 16.13)**	**126**	**57.53**

### Mosquito surveillance

#### Detection of JEV from mosquitoes

A total of 87,201 mosquitoes belonging to more than 4 species (*Culex tritaeniorhynchus*, *Culex pipiens pallens*, *Anopheles sinensis*, and others) were collected from livestock sheds in 6 surveillance sites (Fig 2) during every June to August from 2015–2018. Among them, 76,932 (88.22%) were *Culex tritaeniorhynchus*, 3,696 (4.24%) were *Anopheles sinensis*, 3,284 (3.77%) were *Culex pipiens pallens*, and 3,289 (3.77%) were other species, including *Armigeres subalbatus*, *Aedes albopictus*, *Culex inatomii*. Xianju County played an important role in mosquito surveillance from 2006 to 2015, and Yiwu County was the only site that conducted mosquito surveillance annually from 2015 to 2018. The remaining 4 counties were newly established in the last two years and contributed strongly to JE surveillance.

**Fig 3:**
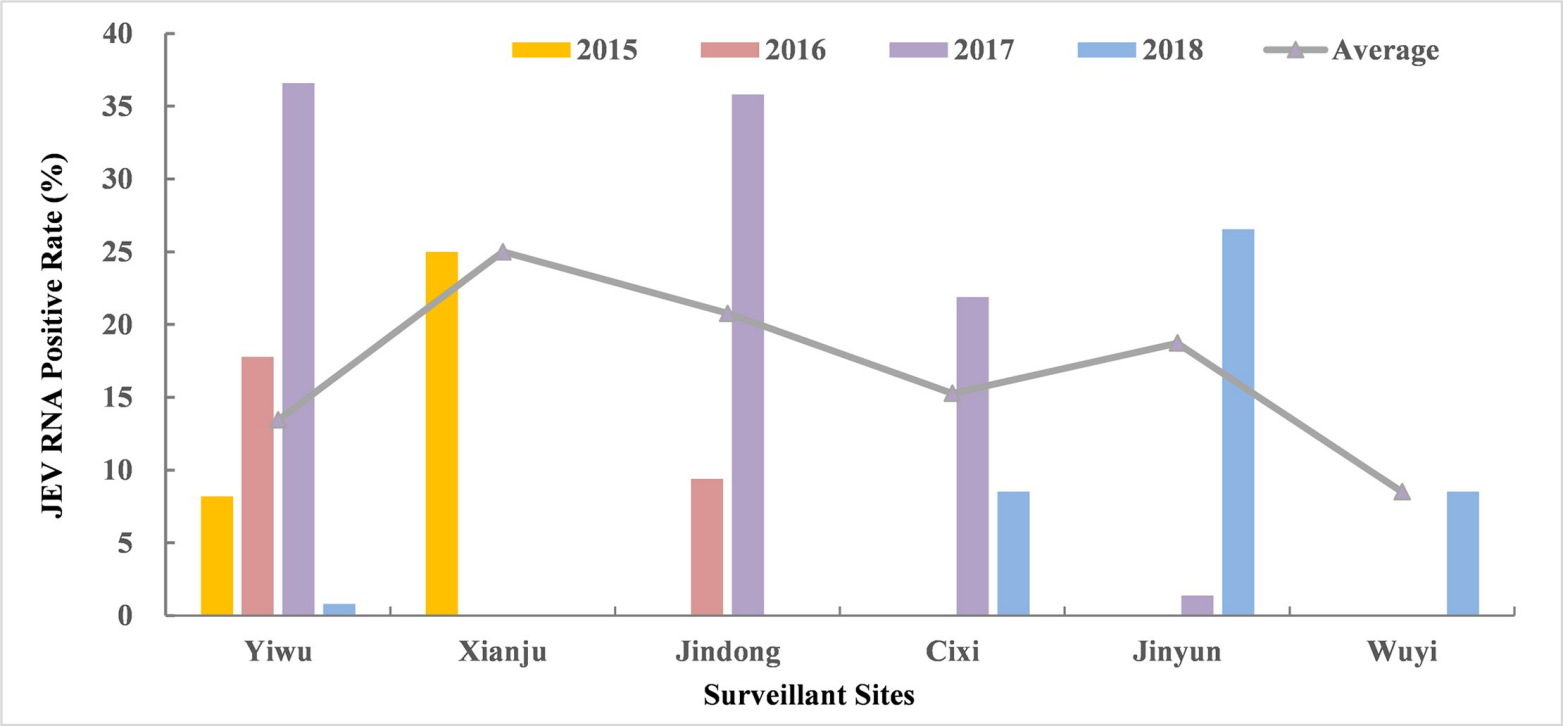
JEV RNA positive rate among *Culex tritaeniorhynchus* of mosquito surveillance sites in Zhejiang, 2015–2018.

Mosquitoes were classified into 1,495 pools according to morphological characteristics, of which 230 pools were JEV positive by RT-PCR amplification of E gene. The annual JEV positive rate among every site varied largely from the lowest of 0.73% (2018 Yiwu) to the highest of 36.31% (2017 Yiwu), and the rates of the same site for the adjacent two years also fluctuated extremely (Table 4, Fig 3). The overall positive rates of JEV RNA were respectively 15.38% and 16.33% among the total and the *Culex tritaeniorhynchus* during 2015–2018. The highest annual JEV positive rate among the *Culex tritaeniorhynchus* occurred in 2017 with 26.81%, and a relatively low rate occurred in 2018 with 10.23%. The infection rate according to bias-corrected MLE of JEV in the *Culex tritaeniorhynchus* with 95% confidence intervals (CI) were 1.56 (0.91, 2.52), 2.36 (1.52, 3.52), 5.65 (4.67, 6.78) and 1.77 (1.35, 2.27) per 1,000, respectively.

**Table 4:** Sampling and detection of JE mosquito surveillance in Zhejiang from 2015–2018.

Year	Sites	Total				*Culex tritaeniorhynchus*			
		No. of Individuals	No. of Pools	No. of Positive Pools	RNA Positive Rate (%)	No. of Individuals	No. of Pools	No. of Positive Pools	RNA Positive Rate (%)
**2015**	**Total**	**13904**	**190**	**19**	**10.00**	**10129**	**130**	**15**	**11.54**
	Xianju	5945	78	10	12.82	2385	24	6	25.00
	Yiwu	7959	112	9	8.04	7744	110	9	8.18
**2016**	**Total**	**11447**	**170**	**22**	**12.94**	**10008**	**154**	**22**	**14.29**
	Yiwu	6197	100	16	16.00	5208	90	16	17.78
	Jindong	5250	70	6	8.57	4800	64	6	9.38
**2017**	**Total**	**23789**	**427**	**113**	**26.46**	**22827**	**414**	**111**	**26.81**
	Yiwu	5674	168	61	36.31	5426	164	60	36.59
	Jindong	5320	85	29	34.12	5100	81	29	35.80
	Cixi	7794	101	22	21.78	7300	96	21	21.88
	Jinyun	5001	73	1	1.37	5001	73	1	1.37
**2018**	**Total**	**38061**	**708**	**76**	**10.73**	**33968**	**557**	**57**	**10.23**
	Cixi	4820	95	8	8.42	4700	94	8	8.51
	Jinyun	14100	282	62	21.99	12500	162	43	26.54
	Yiwu	16341	275	2	0.73	14568	254	2	0.79
	Wuyi	2800	56	4	7.14	2200	47	4	8.51
**Total**	**87201**	**1495**	**230**	**15.38**	**76932**	**1255**	**205**	**16.33**

#### Sequence Analysis on E Gene

Homologous comparison and sequence analysis of E gene were conducted among the 145 samples, including 139 JEV-positive samples (GI) derived in 2015–2018, 3 GIII strains derived in 1982–1983, the live attenuated vaccine (SA 14-14-2) strain, and 2 known virulent strains (P3 and Beijing-1). Homologous comparisons indicated that the 139 sequences of E gene derived from 6 surveillance sites maintained a high level of stability with each other at the nucleotide (range: 98.8%-99.8%) and amino acid (range: 99.4%-100%) levels, but a relatively lower sequence similarity to 1982–1983 GIII strains (87.2%-87.6% at the nucleotide level and 98.6%-98.8% at the amino acid level) and SA 14-14-2 strain (84.9%-86.1% at the nucleotide level and 96.7%-97.2% at the amino acid level). The nucleotide sequence divergences within the genotype from Zhejiang Province were only 0.2%-2.2% (GI) and 0%-0.9% (GIII). The nucleotide sequence divergences between GI and GIII from Zhejiang Province were 12.4%-12.8%.

Sequence analysis of E gene indicated that mainly 14 amino acid residues in the 139 newly detected E gene samples differed from the live attenuated vaccine SA 14-14-2 strain (Table 5), including eight key amino acid residues related to virulence and virus attenuation (E107F→L, E138K→E, E176V→I, E177A→T, E264H→Q, E279M→K, E315V→A and E439R→K). The other eight amino acid residues were found to be different between P3 strain and the 139 samples (E76M→T, E129T→M, E222A→S, E227P→S, E306G→E, E327S→T, E366A→S, E408L→S). As expected, no mutation was observed at the 8 key amino acid residues between Beijing-1 strain and the 139 samples, another eight amino acid residues were detected to be different (E129T→M, E132S→P, E222A→S, E227P→S, E327S→T, E366A→S, E397Y→H, E473I→V). Compared with the three isolated GIII strains in Zhejiang Province, 4 amino acid residues in 139 JEV-positive GI samples showed differences (E129 T→M, E222 A→S, E327S→T, E366A→S).

**Table 5:** Comparison of the amino acid residues of E gene among 145 samples.

Amino Acid Residue	Domain	SA 14-14-2	P3	Beijing-1	3 GIII strains^a^	139 GI samples^b^
**E76**	II	T	M	T	T	T
E107*	II	F	L	L	L	L
**E129**	II	T	T	T	T	M
**E132**	II	P	P	S	P	P
E138*	I	K	E	E	E	E
E176*	I	V	I	I	I	I
E177*	I	A	T	T	T	T
**E222**	II	A	A	A	A	S
**E227**	II	S	P	P	S	S
**E244**	II	G	E	E	E	E
E264*	II	H	Q	Q	Q	Q
E279*	II	M	K	K	K	K
**E306**	I	E	G	E	E	E
E315*	III	V	A	A	A	A
**E327**	III	S	S	S	S	T
**E366**	III	A	A	A	A	S
**E397**	III	H	H	Y	H	H
**E408**	III	S	L	S	S	S
E439*	Non	R	K	K	K	K
**E447**	Non	D	G	G	G	G
**E473**	Non	V	V	I	V	V

Note

*indicates the eight key amino acid residues related to neuroinvasiveness and neurovirulence attenuation in E gene.

^a^ indicates the 3 isolated GIII JEV strains in Zhejiang Province in 1982–1983.

^b^ indicates the 139 JEV-positive sequencing samples (GI) in Zhejiang Province in 2015–2018.

Though the 139 samples maintained high stability on E gene sequence, mainly 8 amino acid mutations (≥ 3 samples) were observed among them, which were distributed in three domains and non-structural domain (Table 6). The amino acid residue with the highest mutation rate (7.91%) was E89 from domain II (S→N, 11 samples), followed by E434 from non-structural domain (F→L, 8 samples, mutation rate of 5.76%) and E397 from domain III (H→Y, 7 samples, mutation rate of 5.04%).

**Table 6:** Comparison of the amino acid residues of E gene among the 139 JEV-positive sequencing GI samples.

Amino acid residue	Domain	Mutation	No. of mutation samples	Mutation Rate (%)
**E89**	Ⅱ	S→N	11	7.91
**E434**	Non	F→L	8	5.76
**E397**	III	H→Y	7	5.04
**E261**	II	G→A	5	3.60
**E36**	I	N→S	4	2.88
**E486**	Non	A→V	4	2.88
**E86**	II	A→V	3	2.16
**E369**	III	K→R	3	2.16

#### Phylogenetic analysis on E Gene of JEV

**Fig 4:**
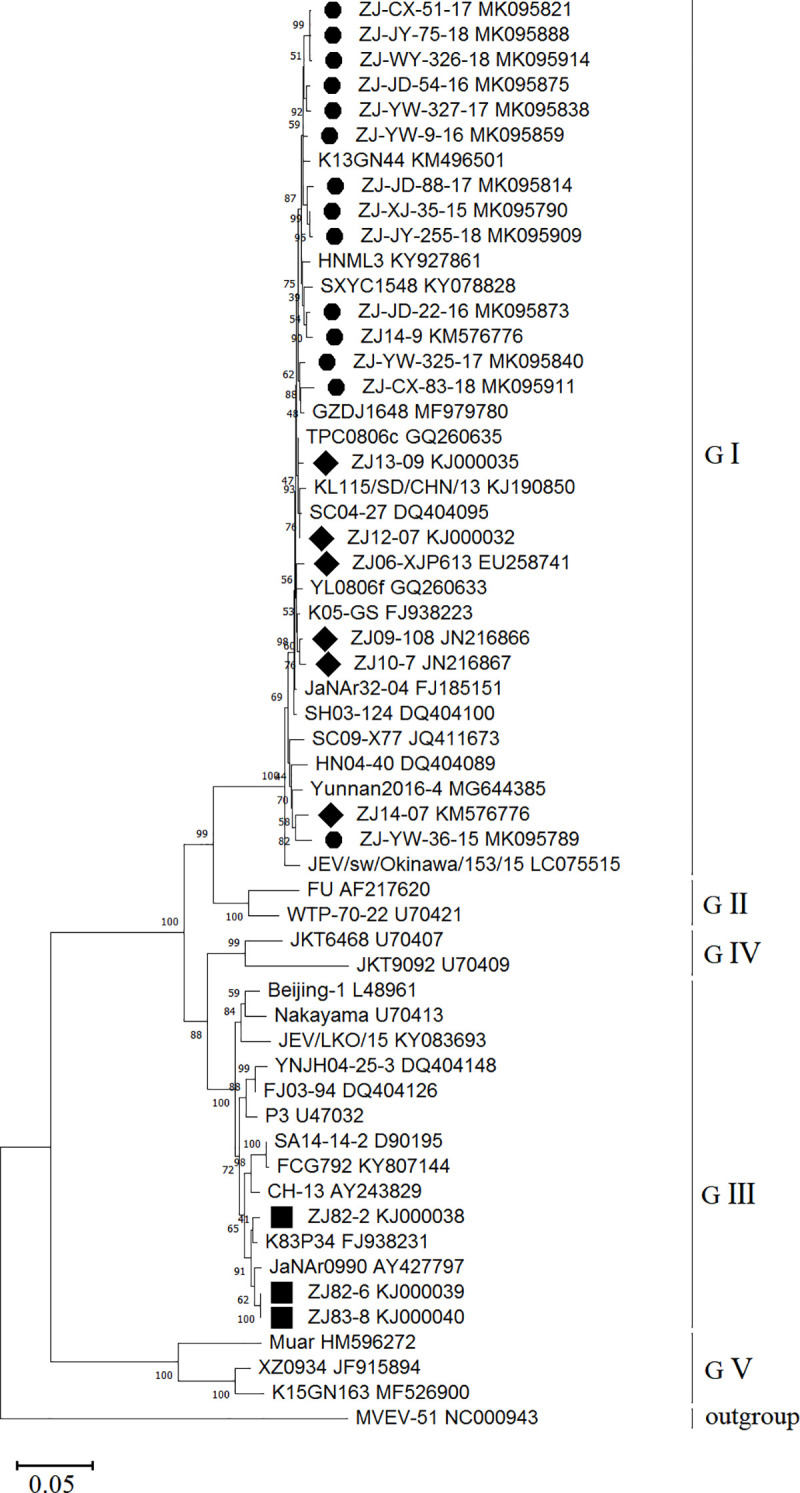
Phylogenetic tree on 1500-nt envelope gene of Japanese encephalitis virus strains. A total of 57 selected strains were involved in phylogenetic analysis, including 13 E gene samples (2015–2018), 10 strains (7 strains from 2006–2014 and 3 strains from 1982–1983) derived from Zhejiang Province and 34 strains derived from other provinces in China (Beijing, Fujian, Henan, Hunan, Guangxi, Gansu, Guizhou, Shandong, Shanghai, Shanxi, Sichuan, Taiwan, Tibet, Yunan) or abroad. The sequences of Zhejiang strains isolated in 1982–1983 and 2006–2018 are marked in black rhombus and circle, respectively. The virus name and Genbank accession number are noted. Phylogenetic analysis was performed by the maximum-likelihood method using MEGA 7.0 software package. Labels of strains conform to the following format: Strain name (including region and year of isolation) and GenBank accession number.

The 1,500-nt E gene were successfully amplified in 139 JEV-positive sequencing samples derived from mosquitoes in Zhejiang Province during 2015–2018, which had been submitted to GenBank (MK095778-MK095916). A total of 57 selected strains were involved in phylogenetic analysis (Fig 4), including 23 E gene samples derived from Zhejiang Province, and 34 strains belonging to different genotypes derived from other provinces in China or abroad. All the 57 strains were clustered into five genotypes: I, II, III, IV and V. Phylogenetic analysis indicated that the Zhejiang strains were classified into two genotypes. The strains isolated in 1982–1983 were clustered into GIII and those isolated in 2006–2018 were all clustered into GI. Selected strains isolated from other regions in China or abroad in recent years all belong to GI. Zhejiang strains derived during 2016–2018 were genetically similar to the South Korea strain (KM496501) in 2013, while a relatively long distance was observed between the Zhejiang strains and the Japan strain (LC075515) in 2015.

## Discussion

Due to the high-quality childhood JE vaccination program and dramatically improved hygienic conditions, the situation of JE in Zhejiang Province was well controlled. However, the burden of Japanese encephalitis was still heavy due to the high rate of disability and mortality. In our study, 28.30% of 53 JE cases had long-term sequelae when followed-up at the sixth month after onset. Similar studies of JE prognosis conducted in China [25], Malaysia [26] and Cambodia [27] all reflected the heavy burden of JE on society, as well as families. Encouragingly, according to the morbidity and mortality weekly report on JE Surveillance and Immunization in Asia and Western Pacific Regions in 2016 [28], 22 of the 24 countries (92%) with JEV transmission risk conducted JE surveillance, which was only 75% in 2012. However, the JE immunization program has not fully covered, with the percentage of countries increasing from 46% in 2012 to 50% in 2016.

It was challenging that there was a shift to a greater proportion of adult cases in China, especially in the northern area of high latitude, which was previously low endemic. The incidence of JE under 15 years of age decreased significantly [29]. Most adult cases were middle-aged farmers who had never been vaccinated or had no chance of being naturally infected. As the global warming and rainfall increased, mosquitoes were more active and breed longer. The frequency of circulation and spread of JEV was remarkably increased, leading to a higher risk of JEV exposure [30]. JE prevention and control among adults has become an urgent problem due to the lack of a uniform and authoritative immunization strategy in China. Stronger emphasis should be placed on changing livestock farming style and enhancing mosquito control. The epidemiology trend was also detected in other regions [31–39] regardless of whether JE burden was high or whether the immunization program was fully implemented, such as Bangladesh [31], South Korea [32–33], Japan [34–35], and India [39].

Our study indicated that JE neutralizing antibody positive proportion was relatively lower in the <25-year-old age groups (except for the 1-3-year-old group). For the <1-year-old age group, GMT was highest at birth and significantly decreased with increasing month-age. The outcomes were consistent with the serosurvey conducted in 2013–2014 in Zhejiang Province [18]. It is suggested that the maternal transferred JE antibody level was under the threshold of protection (<1:10) even at birth, not to mention the follow-up. Therefore, it is essential to obtain a timely dose of JE vaccine at 8 months of age or even earlier if necessary. In addition, the seropositive proportion against P3 strain of 7-14-year-old group was only 22.12%, indicating that a booster dose at 6 years old may be needed. In our study, adults had a significantly higher GMT, mainly due to a wider range of activities and a higher chance of being bitten, which implied that the natural circulation of JEV was active and most were latent infections. On the other hand, the lower level of neutralizing antibodies in young age groups indicated that the immune barrier had not been fully established among children and adolescents, which was in accordance with the age-specific incidence of JE in Zhejiang Province. Although there was a tendency toward a greater proportion of adult JE cases, the age-specific incidence of the 0-4-year-old group was highest during 2015–2018. Despite Zhejiang Province started to use JE vaccine as early as in 1953, the vaccination rate was too low to establish herd immunity due to the turbulent society and underdeveloped economy to produce enough vaccine. In addition, the vaccine types and immunization doses differed greatly as time went on. In the early 1960s, one dose of inactivated mouse brain vaccine was used in limited area with highly adverse reaction rate. Then, live attenuated JE vaccine (JEV-L) was applied in 1989 and the vaccination rate among the ≤ 6-year-old population could not be guaranteed until the national Expanded Program on Immunization in 2008 in China. After several years of hard work, the overall incidence of JE in Zhejiang Province sharply declined. Since the last dose of JE vaccine for children is administered at 2 years old (live attenuated JE vaccine) or 6 years old (inactivated JE vaccine) in Zhejiang Province, the reason for seropositivity drop among 7–14 years old group maybe a combined effect of attenuation of antibody level and lack of natural infection due to the limited movement. Similar domestic serosurveys conducted in Yangzhou City [40] and Tongchuan City [41] both demonstrated lower GMT levels in young age groups. Other studies carried out in Jiangsu Province [42] and Xinyang City [43] showed considerably higher seropositive proportion than ours (67.13% of 5-14-year-old group and more than 82.98% for 7-14-year-old group, respectively). In non-endemic area, such as Tibet [44], the seroprevalence rate was even higher for the < 45-year-old group and only 4.2% for > 45-year-old population, which showed contrary tendency to ours on age-specific seropositive proportion. This was mostly due to increased movement to epidemic areas in younger populations. South Korea had accumulated extensive experience on JE surveillance. They found the overall positive rate among high-risk age groups (≥30 years old) was 98.1%, which was higher than ours (74.53%-77.50%). Differences in rates among different regions might be due to 3 possible reasons: the first was the different nature conditions and living style, including climate, landscape, rice paddy, pig farming and population mobility, which determine the natural infection baseline and significantly influence the circulation intensity of JEV. The second reason was the JE vaccination coverage rate as well as the immunization strategy, including doses, intervals and vaccine types, which played an important role in the establishment of immune barrier. Antibody attenuation and immunogenic persistence are core factors to the seroprevalence of antibody level. The third reason was test methods of neutralizing antibody, as well as the selection on JEV strains. Thus far, PRNT is the most commonly accepted test that can discriminate cross-reacting antibodies in primary flavivirus infections [45]. Some studies adopt ELISA (enzyme-linked immunosorbent assay) to measure the antibody level, which might give a biased outcome.

Regarding to the duration of protection and immunity, our study indicated that 4 or more years after the 2^nd^ dose of JEV-L, the seropositive proportions decreased to 32.5%-37.5% and GMTs decreased to 8.08–9.11, further confirming that a booster dose at 6 years old would be necessary. Currently, two doses of primary hamster kidney (PHK) cell-derived, live attenuated vaccine based on SA 14-14-2 strain (GIII) are commonly used as EPI vaccine for ≤14-year-old populations in China. The waning trend of neutralizing antibody was also observed in Pan JR’s research [18]. To date, limited study has focused on the long-term immunogenicity of JEV-L vaccine, which is only used in a number of Asia countries. A 5-year follow-up of serosurvey in Nepal children with a single dose of JEV-L in 2000 indicated that the seropositive proportion was 89.9% in 2004 and it remained 63.8% in 2005 [46]. Long-term surveillance of duration for protection with different immunization strategies is still needed.

The mechanism of changing genotypes in traditional JE epidemic areas is a hot topic. Our study showed that no mutation was observed at the 8 key amino acid residues related to virulence and virus attenuation on E gene between GI and GIII strains, but another 4 amino acid residues were detected different (E129T→M, E222A→S, E327S→T and E366A→S), which were also partially or entirely detected in researches in Shanghai Province (E222, E327 and E366) [47], Shandong Province (E129, E222, E327 and E366) [48] and Thailand (E222, E327 and E366) [49]. Xiao C [50] suggested that GI strains developed higher viremia titers and longer viremic durations than GIII strains in avian cells and birds, resulting in a more efficient transmission in the bird-mosquito-bird cycle for GI JEV. Schuh AJ [15] found both GI and GIII viruses were circulating in nature, and sampling sites determined the detected genotype. They preferred that the shift trend was due to molecular adaptation at residue E15 and coevolution within the GI E protein alignment, leading to an acquired ability to be active in lower temperature areas. Amino acids on the top area of Domain III, where E327 and E366 were located are related to virulence, neurotropism and attenuation of flaviviruses [51], which implied that the two residues might have an effect on the tendency of GI to replace GIII. Currently, some articles have confirmed several significant amino acid residue mutations on E gene. For example, mutations at residues L107F, E138K, I176V, T177A, E244G, Q264H, K279M, A315V, S366A and K439R are essential and important for neurovirulence attenuation [52], especially L107F, E138K [53] and M279K [54]. Mutation at residue S123R increase pathogenicity [55]. Regarding to the amino acid residues at E337, E360 and E387, located in major antigenic loops in JEV domain III, are suggested to be the antigenic sites in relation to antibody neutralization [6,56], no divergence was detected in our study among Zhejiang JEV GI strains, GIII strains and the vaccine strain (SA 14-14-2). Therefore, the present widely used JE vaccine based on GIII strains may still provide protection and prevention for current GI strain prevalence. However, further cross-protective capacity tests are needed.

The present work has several limitations. First, random sampling was failed in enrollment of antibody level surveillance, especially for the <1-year-old group, which might not truly reflect the population immunity level. Second, PRNT was recommended by WHO to be the most accepted test to measure neutralizing antibody level, while our study used microseroneutralization assay. Third, isolated JEV strains derived from mosquitoes during 2015–2018 were limited, thus amplified 139 JEV-positive sequencing samples were used for E gene analysis. Last, mosquito collection was repeated annually in only one county (Yiwu), making it difficult to comment on trends of JEV RNA positivity over 2015 to 2018.

In summary, our study discovered that the maternal transferred JE antibody levels were below the threshold level of seropositive (<1:10), even at birth. The seropositive proportions were relatively lower in the 4-24-year-old group. A significant decreasing trend in GMT with age was detected not only in the under 1-year-old population (unvaccinated), but also in the 1-14-year-old group (vaccinated). GMTs against P3 strain decreased to below 1:10 after 4 or more years past the 2^nd^ dose of JEV-L. JEV strains isolated from mosquitoes during 2015–2018 all belonged to GI with infection rates of 1.56, 2.36, 5.65 and 1.77 per 1,000 mosquitoes, respectively, suggesting a high risk of human infection in Zhejiang Province. No divergence on important antigenic sites of the E gene was detected between the Zhejiang JEV strains and the SA 14-14-2 strain, while the eight critical amino acid residues responsible for neuroattenuation were completely different. Thus, from an administrative perspective, further studies can be emphasized on the evaluation of JE vaccination coverage of target population, as well as the condition of cold-chain system. In addition, molecular pathology and toxicology tests, protective efficacy analysis of current JE vaccines to five genotypes of JEV, should be enhanced to contribute to the development of JE vaccines. Surveillance of provincial and national JE serological survey in healthy populations with different sequential immunization schedule can be conducted to provide evidence for reformation on JE immunization strategy.

## Supporting information

S1 Checklist STROBE checklist. (DOC)

S1 Table Supplementary Data. **Microseroneutralization test results.** A total of 429 infants (under 1 year old) and 761 participants (over 1 year old) were enrolled, a 2ml blood sample was drawn and tested by microseroneutralization test. P3(GIII) strain was neutralized by serum samples from participants.
(XLS)

S2 Table Supplementary Data. **Details of JEV strains involved in Phylogenetic Analysis in this study.** A total of 57 selected strains were involved in phylogenetic analysis, including 23 samples derived from Zhejiang Province, and 34 strains derived from other provinces in China or abroad. Isolated year, source, region, country, genotype and GenBank accession number were listed for each sample.
(XLSX)

S3 Table Supplementary Data. **Sequence comparison of amino acid differences in envelop protein of JEV strains isolated in Zhejiang Province and other related strains.** A total of 500 amino acids in E gene of 145 JEV samples were listed, analyzed by MEGA 7.0 software package. The virus name and GenBank accession number were noted. “.” means the same type of amino acid as the first strain (SA 14-14-2 strain).
(XLS)
